# Severe Sinus Dysfunction in the Context of Chronic Lithium Intoxication and Poorly Controlled Hypothyroidism

**DOI:** 10.7759/cureus.55127

**Published:** 2024-02-28

**Authors:** Carlos M Ardila, Alejandro López-Valencia, Daniel González-Arroyave

**Affiliations:** 1 Basic Sciences, University of Antioquia, Medellin, COL; 2 Emergency Medicine, Hospital San Vicente Fundación, Rionegro, COL; 3 Surgery, Universidad Pontificia Bolivariana, Medellin, COL

**Keywords:** emergency medicine, hypothyroidism, intoxication, lithium, sinus node dysfunction

## Abstract

Cardiotoxicity associated with lithium is not a common event; however, it is potentially life-threatening, manifesting electrocardiographically with sinoatrial blocks, high-degree atrioventricular blocks, QT prolongation, and ventricular tachyarrhythmias. This case report presents a patient with severe sinus dysfunction in a clinically severe presentation secondary to cardiogenic shock. The patient sought medical attention for a one-week history of non-anginal chest pain, dizziness without syncope, generalized weakness, and somnolence progressing to bedridden status in the days preceding hospital admission. Laboratory findings revealed elevated blood levels of lithium and thyroid-stimulating hormone (TSH), along with concomitant Acute Kidney Injury Network (AKIN) II acute kidney injury. Subsequently, the patient was admitted to the intensive care unit, where persistent extreme sinus bradycardia of 30 bpm (beats per minute) with sinus pauses without ischemic changes was observed. The patient received supportive treatment, including renal replacement therapy, resulting in complete recovery of hemodynamic status without the need for long-term cardiac conduction devices.

## Introduction

Since the 19th century, lithium carbonate has been used in the treatment of depression and mania. In the 1930s, it was employed as a salt substitute in heart failure patients; however, due to poisoning manifestations, its use had to be discontinued [1]. Currently, it is considered the most effective long-term therapy for the treatment and prevention of relapse in bipolar affective disorder, constituting its primary indication [2]. It is also widely recommended for acute manic episodes and suicide prevention [3]. In the United States, according to the American Association of Poison Control Centers, around 6000 incident cases of lithium poisoning were reported in 2020, with a severe clinical presentation observed in approximately 3% of cases, primarily resulting from suicide attempts in individuals over 20 years old [4].

Blood lithium levels have a narrow therapeutic range, making them susceptible to various types of intoxication. Several risk factors are recognized for pathological lithium accumulation and subsequent toxicity development, including age, renal disease, thyroid dysfunction, and concurrent use of nonsteroidal anti-inflammatory drugs (NSAIDs), angiotensin-converting enzyme inhibitors (ACEIs), angiotensin II receptor antagonists (ARBs), beta-blockers, and diuretics [5]. Clinical manifestations vary widely and occur in variable degrees.

Cardiotoxicity associated with lithium is not a frequent event; however, it is potentially life-threatening, manifesting electrocardiographically with sinoatrial blocks, high-degree atrioventricular blocks (AVB), QT prolongation, and ventricular tachyarrhythmias.

This case emphasizes the presence of persistent extreme sinus bradycardia and sinus pauses without escape rhythm despite bradycardia, which did not improve completely until renal replacement therapy was initiated, leading to the removal of lithium from the blood. The objective of this case is to present an unusual electrocardiographic finding secondary to lithium carbonate intoxication, accentuated by poor thyroid control and concurrent use of prohibitive medications.

## Case presentation

A 76-year-old female, hailing from a rural area, with a personal history of remitted breast carcinoma, currently undergoing oral chemotherapy with letrozole (2.5 mg daily), managing primary hypothyroidism with levothyroxine supplementation (75 µg daily), controlling primary hypertension with losartan (50 mg every 12 hours), and managing bipolar affective disorder with lithium carbonate (600 mg daily), without follow-up for the past four years. The patient sought medical attention due to a one-week history of non-anginal chest pain, dizziness without syncope, generalized weakness, and drowsiness, progressing to bedridden status in the days before hospital admission. Initial evaluation at a low-complexity hospital documented a heart rate (HR) of up to 35 beats per minute (bpm). Progressive doses of atropine up to 3 mg were administered without improvement, prompting the initiation of transcutaneous temporary cardiac pacing with the following parameters: HR of 60 bpm and 40 milliamps (mA), along with adrenaline infusion without improvement. Subsequently, the patient was immediately referred to a higher-complexity center. Table 1 displays the clinical laboratory parameters of the patient post-admission to a high-complexity hospital.

**Table 1:** Clinical laboratory values of the patient after admission to a high-complexity hospital.

Parameter	Patient value	Normal reference value
Heart rate	38 beats per minute	60-100 beats per minute
Mean arterial pressure	100/64 mmHg	120/80 mmHg
Thyroid-stimulating hormone (TSH)	19 mg/dL	1-4.4 mg/dL
Free normal thyroxine (T4L)	1.0 ng/dL	0.89-1.76 ng/dL
Serum lithium levels	1.78 mEq/L	0.6-1 mEql/L

**Figure 1:**
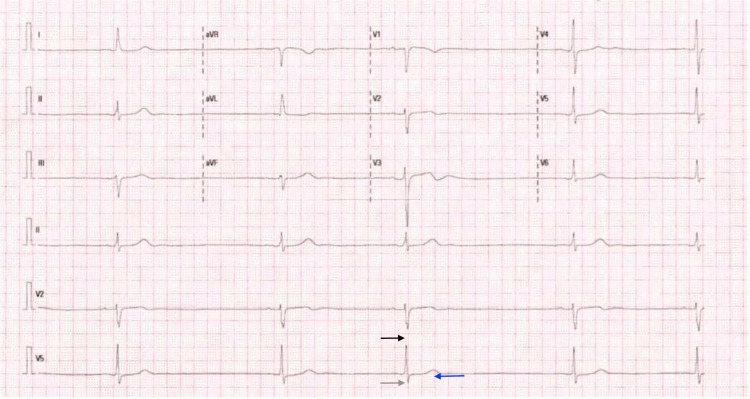
Twelve-lead Electrocardiogram showing sinus bradycardia rhythm, heart rate of 26 beats per minute, PR interval of 182 ms (black arrow), sinus pause without escape rhythm, irregular RR interval, QRS duration of 106 ms (gray arrow), QTc interval of 525 ms (blue arrow), left axis deviation of the QRS complex, poor progression of the R wave in precordial leads, no alterations in repolarization, and not supra or infra-ST segment depression.

Upon admission, she was hemodynamically unstable (low HR and mean arterial pressure) with decreased alertness and a prolonged capillary refill of four seconds. Adjustments were made to 120 mA, and adrenaline infusion was continued up to 0.1 mcg/kg/minute, without clinical improvement, leading to orotracheal intubation. Initial paraclinical studies showed stable acid-base status, normal serum oxygen levels, lactate, ions, and glucose, a normal hepatic biochemical profile, and negative myocardial injury. However, acute kidney injury AKIN II, poorly controlled primary hypothyroidism with increased TSH, free normal thyroxine (T4L), and elevated serum lithium levels were evident. Upon admission to the intensive care unit, extreme persistent sinus bradycardia of 30 bpm with sinus pauses without ischemic changes was observed (Figure 1).

Interestingly, non-invasive cardiac monitoring methods frequently revealed sinus pauses without escape rhythm despite extreme bradycardia. Temporary transvenous cardiac pacemaker placement was decided for treatment. Based on the clinical context and paraclinical findings, the diagnosis of chronic lithium intoxication with severe neurological and cardiovascular manifestations was established. Therefore, intermittent hemodialysis therapy was initiated for three days, accompanied by intravenous isotonic fluid infusion and discontinuation of lithium therapy. In the following 72 hours, the control serum lithium level was at 0.3 mmol/L, with complete recovery from neurological deficit and cardiac conduction disorder. Successful extubation was achieved, and the temporary pacemaker was removed, leading to hospital discharge after four days. In the outpatient psychiatry consultation, the diagnosis of bipolar affective disorder was ruled out, and management for anxiety disorder with 75 mg of pregabalin at night was initiated. The patient is currently asymptomatic.

The patient has given informed consent, authorizing the reporting of case information, including images, management, and results, for academic purposes.

## Discussion

The word "lithium" derives from "litos," a Greek word meaning stone. This uncharged metal has been used therapeutically as a monovalent cation since the 19th century [5,6].

In the context of chronic lithium toxicity, the patient maintains a stable body load of lithium, and poisoning occurs when some factor disrupts the therapeutic balance, either by increasing absorption or, more commonly, by decreasing elimination. Cardiotoxicity associated with lithium is not a common event; however, when present, it represents a potentially life-threatening acute event [6].

The therapeutic index of lithium is narrow, and the generally accepted serum range is concentrations between 0.6 and 1.2 mmol/L [7]. Concentrations exceeding 1.5 mmol/L pose a high risk of severe toxicity. The central nervous system is the most significant site of lithium toxicity, whose development is directly related to the type of exposure pattern. In cases of acute poisoning, neurological manifestations are unlikely, unlike patterns of acute-on-chronic or chronic exposure, which can range from mild symptoms such as tremors and weakness to severe conditions like non-convulsive epileptic states or stupor. Symptoms of cerebellar compromise, extrapyramidal manifestations, and neuromuscular signs (myoclonus or hyperreflexia) are also described. The literature mentions a concept that unifies these symptoms into a syndrome called lithium-induced neurotoxicity (SILENT) [5].

Lithium has a low volume of distribution and low protein binding, allowing it to freely distribute in total body water, undergoes minimal metabolism, and is almost entirely eliminated by the kidneys, with a small amount excreted in feces. Therefore, it is an excellent dialyzable medication in cases of intoxication or severe manifestations [8].

The side effects of lithium on cardiac tissue range from benign to life-threatening, and they may occur even at therapeutic levels [9]. Among the most common findings are alterations in repolarization and sinus bradycardia; however, severe electrocardiographic complications are uncommon. This was demonstrated in an observational cohort of 502 patients over five years in California, USA, where only around 1% experienced a life-threatening cardiac complication [10]. In the present case, clear symptomatic severe sinus dysfunction was observed, with suppression of the sinus node depolarization, without improvement despite non-electric pharmacological stimulation. Cardiotoxic effects have been documented in animal models, particularly those related to sodium channel blockade [11]. Lithium can also alter the depolarization function of the sinus node by interacting with hyperpolarization-activated cyclic nucleotide-gated (HCN) pacemaker channels and/or the sodium-calcium exchanger. It is important to emphasize that cardiac sodium channels are responsible for the pacemaker function of the sinus node, as they are expressed both around and within the sinus node tissue itself [9]. In this line of thought, when there is a blockage of these channels, it leads to a failure in impulse generation or a slowing of conduction to the adjacent atrial myocardium, affecting the pacemaker activity of sinus node cells.

Another key element observed in the current case was the concomitant presence of poorly controlled hypothyroidism, with TSH levels nearly five times higher than the upper normal limit. The prevalence of hypothyroidism in bipolar disorder patients not treated with lithium is between 9 and 10%, compared to 28-32% in treated patients. Therefore, there is a higher prevalence of thyroid dysfunction when exposed to lithium carbonate [12]. The effects on cardiac conduction in the presence of this alteration are well-known. When combined with the effects already mentioned in cases of lithium intoxication, both circumstances can lead to serious electrocardiographic manifestations such as severe sinus bradycardia, sinus arrest, and sinoatrial blocks [13].

To reduce the inherent risks of lithium therapy and, in turn, practice better medical care, it is essential to identify the right patient to initiate this treatment. Regarding the reported case, several significant risk factors were identified, which should have been considered before starting treatment or during respective follow-ups. These include age, concomitant use of losartan, and the absence of monitoring for renal and thyroid function [5]. As mentioned earlier, drugs from the ARB II family and alterations in renal function decrease lithium excretion at the renal level, being the primary elimination source. Hence, the importance of the sine qua non condition of periodic renal function follow-ups to anticipate any deterioration, which lithium per se can trigger [13], and thus prevent conditions that predispose pathological accumulation in the blood beyond therapeutic lithium levels. Monitoring at the initiation of lithium therapy and periodic assessment of TSH and serum creatinine levels are recommended to avoid such outcomes [3]. Moreover, some medications, such as sodium bicarbonate or acetazolamide, may be given to help increase the elimination of lithium from the body [1,2,7].

Several similar cases of severe cardiac conduction disorders in the context of lithium use, such as complete AV block [14], Mobitz II second-degree AV block [15], symptomatic sinus bradycardia [16], and second-degree sinoatrial block [16], have been reported in the medical literature. Sometimes, these cases occurred even with lithium blood levels within therapeutic ranges [17], and each required pacemaker implantation, either temporarily or permanently. In one of these cases, the presentation included elevated lithium levels and acute kidney injury with concomitant NSAID use, requiring, as in the present case, dialysis therapy for comprehensive management and complete resolution of the condition [16]. Only in two reports was the presence of poorly controlled hypothyroidism evident [12,18], in the context of lithium consumption and severe electrical conduction disorders. In one of them, the initiation of thyroid hormone supplementation alone was sufficient for improvement [18].

As strengths in managing the present case, timely referral to a higher-complexity center is highlighted, with correct initial management focused on the patient with bradycardia and signs of hypoperfusion. The major differential diagnoses that could more frequently explain the patient's clinical state were appropriately ruled out. Once the cause of neurological and cardiovascular manifestations was determined, the management was defined, leading to a successful recovery of the patient.

## Conclusions

The case illustrates the clinical complexity of chronic lithium intoxication, involving not only neurological manifestations but also severe cardiac conduction disorders. In this case, it is confirmed that lithium-associated cardiotoxicity is a real risk, even at therapeutic levels, and can manifest with serious complications in cardiac conduction, as seen in the case of severe sinus dysfunction. The need for regular reassessment of renal function is emphasized to anticipate any deterioration, given that lithium can trigger renal complications. Additionally, the importance of regularly assessing TSH and serum creatinine levels during lithium therapy is underscored.
